# Obvious Surface States Connecting to the Projected Triple Points in NaCl’s Phonon Dispersion

**DOI:** 10.3389/fchem.2021.789522

**Published:** 2021-11-15

**Authors:** Li Zhang, Fang Fang, Lixin Cheng, Huiming Lin, Kai Wang

**Affiliations:** ^1^ 'College of Mechanics, Changchun Institute of Technology, Changchun, China; ^2^ Engineering and Technology Center, The Fourth Medical College of Harbin Medical University, Harbin, China; ^3^ School of Chemistry, Harbin Normal University, Harbin, China

**Keywords:** DFT, first-principles calculations, phonon dispersion, surface state, NaCl

## Abstract

With the development of computer technology and theoretical chemistry, the speed and accuracy of first-principles calculations have significantly improved. Using first-principles calculations to predict new topological materials is a hot research topic in theoretical and computational chemistry. In this work, we focus on a well-known material, sodium chloride (NaCl), and propose that the triple point (TP), quadratic contact triple point (QCTP), linear and quadratic nodal lines can be found in the phonon dispersion of NaCl with Fm $\bar{3}$ m type structure. More importantly, we propose that the clear surface states connected to the projected TP and QCTP are visible on the (001) surface. It is hoped that further experimental investigation and verification for these properties as mentioned above.

## Introduction

The recent rapid development in topological materials (Kong and Cui, 2011; Cava et al., 2013; Banik et al., 2018; Kumar et al., 2020; Li and Wei, 2021) makes chemists expect these materials to solve the current challenges in quantum chemistry. A series of topological materials, including topological insulators (Müchler et al., 2012; Bradlyn et al., 2017; Kou et al., 2017; Martín Pendás et al., 2019; Isaeva and Ruck, 2020), spin-gapless semiconductors (Gao et al., 2016; Wang et al., 2016; Wang, 2017; Sun et al., 2020; Yue et al., 2020), and topological semimetals/metals (Zhou et al., 2018a; Schoop et al., 2018; Xu et al., 2020a; Klemenz et al., 2020; Zhao et al., 2020), were predicted by researchers, and some of them are confirmed in experiments. Among them, topological semimetals/metals (Zhong et al., 2016; Zhang et al., 2018; Jin et al., 2019a; Jin et al., 2019b; He et al., 2019; Wang et al., 2020a; Wang et al., 2020b; Xu et al., 2020b; Guo et al., 2020; Jin et al., 2021) always have nontrivial band crossings in their electronic band structures. In addition to their potential applications in technology, they also provide a platform for the study of basic quasiparticles in low cost experiments.

Recently, parallel to electrons, topological concepts have been extended to boson systems such as phonons in crystal materials, classical elastic waves in macroscopic artificial phonon crystals, and magnetic oscillators in magnets. Especially important is that the topological phonon in crystal materials (Jin et al., 2018; Liu et al., 2019; Zheng et al., 2019; Liu et al., 2020; Xie et al., 2021) can provide a potential prospect for regulating heat transfer and electron-phonon interaction. It should be emphasized that the phonon is not limited by the principle of Pauli incompatibility, which means that the experimental detection can be carried out in the whole frequency region of the phonon spectrum.

This work will focus on a famous realistic material, sodium chloride (NaCl). NaCl is with the Fm $\bar{3}$ m type cubic structure and with the space group number 225. The experimental lattice constants of sodium chloride (Abrahams and Bernstein, 1965) are *a = b = c* = 5.62 Å. The Na locates at 4a (0, 0, 0) Wyckoff position, and the Cl locates at 4b (0.5, 0.5, 0.5) Wyckoff position. In this work, using the first principles calculations, we will study the topological signatures of the NaCl’s phonon dispersion. We found that triple points with linear phonon bands dispersion and quadratic phonon bands dispersion coexist in NaCl’s phonon dispersion. More importantly, we will exhibit the interesting phonon surface states of the (001) plane. The authors hoped that the uncovered triple points and their connected surface states in the NaCl phonon system could be confirmed in experiment soon.

## Methods

**FIGURE 1:**
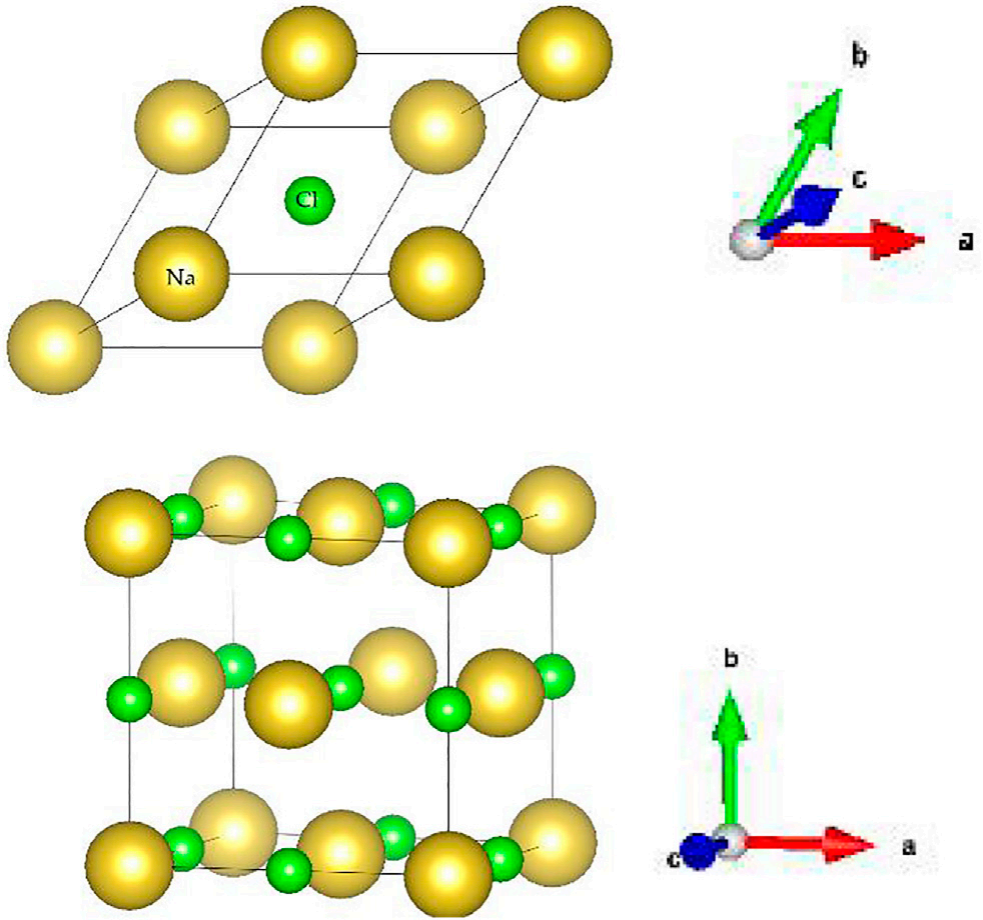
**(Upper)** primitive cell and **(Bottom)** unit cell of NaCl material.

The crystal structure of Fm $\bar{3}$ m NaCl is selected from the Materials Project database (Materials Project, 2021). Some material information, including the magnetic ordering, final magnetic moment, formation energy/atom, band structure, and the phonon dispersion of NaCl, can be found in ref. (Materials Project, 2021). One concludes from ref. (Materials Project, 2021) that NaCl is a nonmagnetic semiconductor with a band-gap value of 5.145 eV. The obtained lattice constants based on first-principle calculations are *a = b = c* = 5.6916 Å, consisting well with the experiment values (Abrahams and Bernstein, 1965). The primitive cell and the unit cell of the NaCl are shown in Figure 1. The yellow and green balls represent the Na and Cl atoms, respectively. This work will focus on the phonon dispersion of NaCl because we would like to uncover its topological signatures. The phonon dispersion of NaCl is determine based on the density functional perturbation theory with the PHONOPY codes (Togo and Tanaka, 2015), and the topological surface properties are constructed by the WANNIERTOOLS package (Wu et al., 2018) based on the phononic Wannier tight-binding Hamiltonian.

### Calculated Phonon Dispersion and the Related Topological Signatures

**FIGURE 2:**
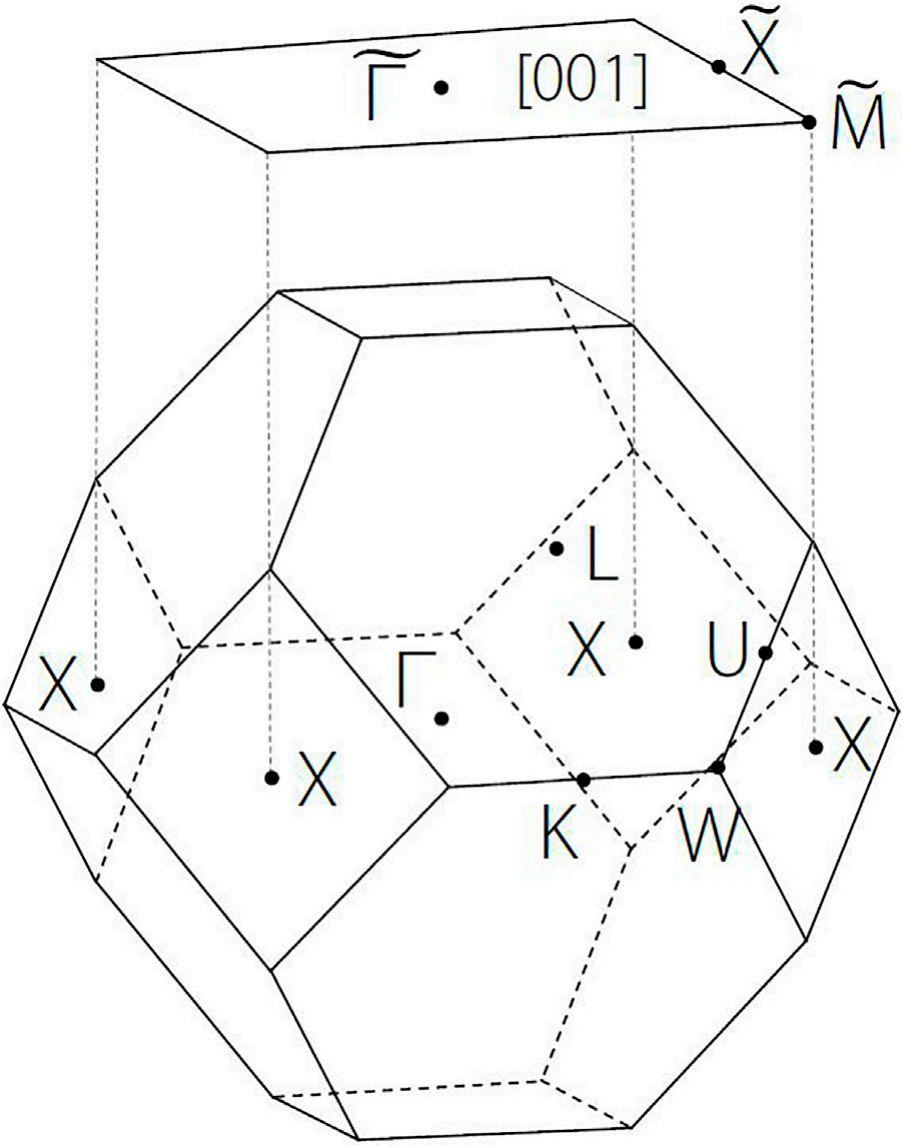
Three-dimensional Brillouin zone (BZ) and the two-dimensional (001) surface BZ. The X, K, W, Y, L, Γ are the symmetry points of 3D BZ. Γ, X, and X points are projected to $\tilde{\Gamma }$ , $\tilde{X}$ , and $\tilde{M}$ points of the (001) surface.

**FIGURE 3:**
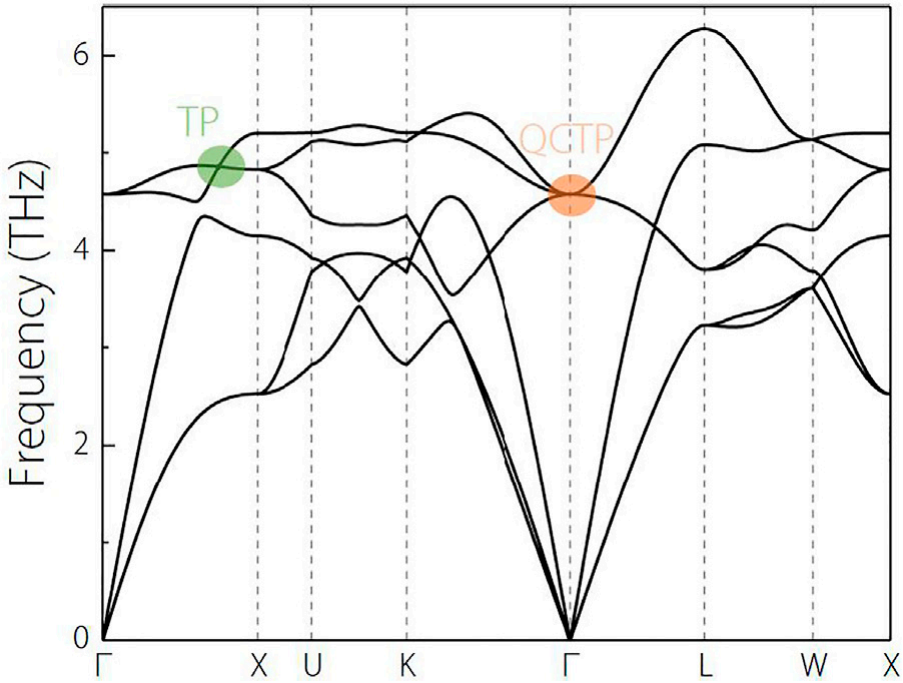
Phonon dispersion of NaCl along the Γ -X-U-K- Γ -L-W-X paths.

In Figure 2, we plotted the three-dimensional BZ and some high symmetry points, X, K, W, Y, L, and Γ . Along the Γ -X-U-K- Γ -L-W-X paths, the phonon dispersion of NaCl is calculated, and the results are shown in Figure 3. During the phonon dispersion calculations, we built a 2 ×2×2 supercell for the NaCl system. From Figure 3, at first glance, one concludes that the NaCl is dynamically stable because the NaCl system has no imaginary frequencies.

Moreover, from Figure 3, one obtains the following information: 1) Along the Γ -X path and in the range of 4–5 THz frequencies, there are one doubly-degenerate phonon band and a non-degenerate phonon band, and these two bands cross at a point (see the green circle in Figure 3) along the Γ -X path. This point along the Γ -X path is a triple point; 2) along the K−Γ path and in the range of 4-5 THz frequencies, one concludes that three phonon bands touched at the Γ point, forming a triple point (see the orange circle region in Figure 3). However, we would like to point out that the triple point on the Γ -X and at Γ are different because the point on the Γ -X is with linear band dispersion and the point at the Γ is with a quadratic band dispersion. Hence, the triple point on the Γ -X is called triple point (TP) (Zhu et al., 2016; Tian et al., 2021), and the triple point at the Γ is usually called quadratic contact triple point (QCTP) (Hu et al., 2019). QCTP features a quadratic band splitting along any direction in momentum space. Along the Γ−L path, one can see that there are also a doubly degenerate band and a non-degenerate band in the range of 4.5–6 THz frequencies.

**FIGURE 4:**
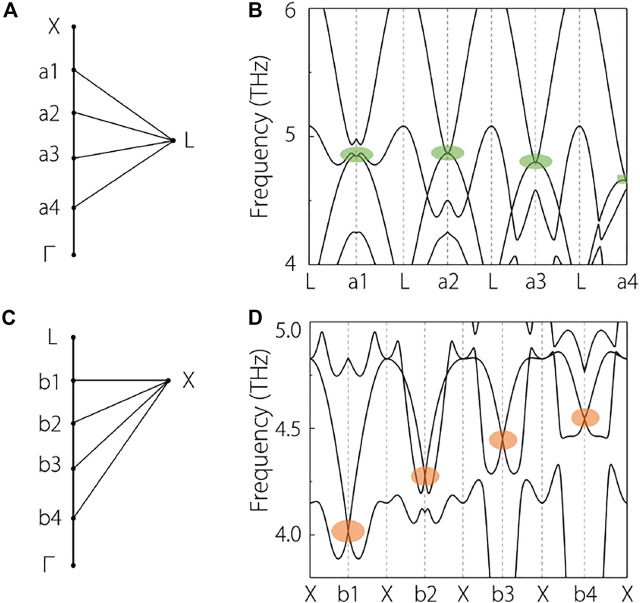
**(A)**, **(C)** some selected symmetry points along the X- Γ and the L- Γ , respectively. **(B)** and **(D)** calculated phonon dispersions along the L-an and X-bn (*n* = 1–4). The linear two-degenerate points and the quadratic two-degenerate points are highlighted with orange and green circles, respectively.

One may wonder whether the doubly degenerate band along the Γ -X (around the TP) and the Γ -L (around the QCTP) paths are the same. In the following, we will answer this question affirmatively. To better answer this question, in Figure 4A,C, we divided the Γ -X (around the TP) and Γ -L (around the QCTP) paths into five parts and selected some more symmetry points. Namely, we selected a1-a4 along the X- Γ and b1-b4 along the L- Γ paths, respectively. The phonon dispersions along the L-an and X-bn (*n* = 1, 2, 3, 4) are shown in Figure 4B,D, respectively. One finds the points at a1, a2, a3 a4 are all with a quadratic band splitting, however, for the points b1, b2, b3, b4, they are with a classic linear band splitting. Hence, the doubly degenerate band along the X- Γ , is composed of doubly degenerate points with linear band splitting, forming a linear nodal line (Zhou et al., 2018b; Chen et al., 2018; Chang et al., 2019; Yan et al., 2019; Li et al., 2020a; Kirby et al., 2020; Meng et al., 2020; Wang and Yang, 2021). The doubly degenerate band along the Γ−L , is composed of doubly degenerate points with quadratic band splitting, forming a quadratic nodal line (Yu et al., 2019; Wang et al., 2020c).

A summary of this section is shown as follow: NaCl phonon hosts a QCTP at the Γ point, a TP along the X- Γ  path, a two-degenerate linear nodal line along the X- Γ path, and a quadratic nodal line along the Γ−L. It is hoped that such rich topological signatures in NaCl can be confirmed in experiment soon.

### Calculated Surface States on (001) Surface BZ

**FIGURE 5:**
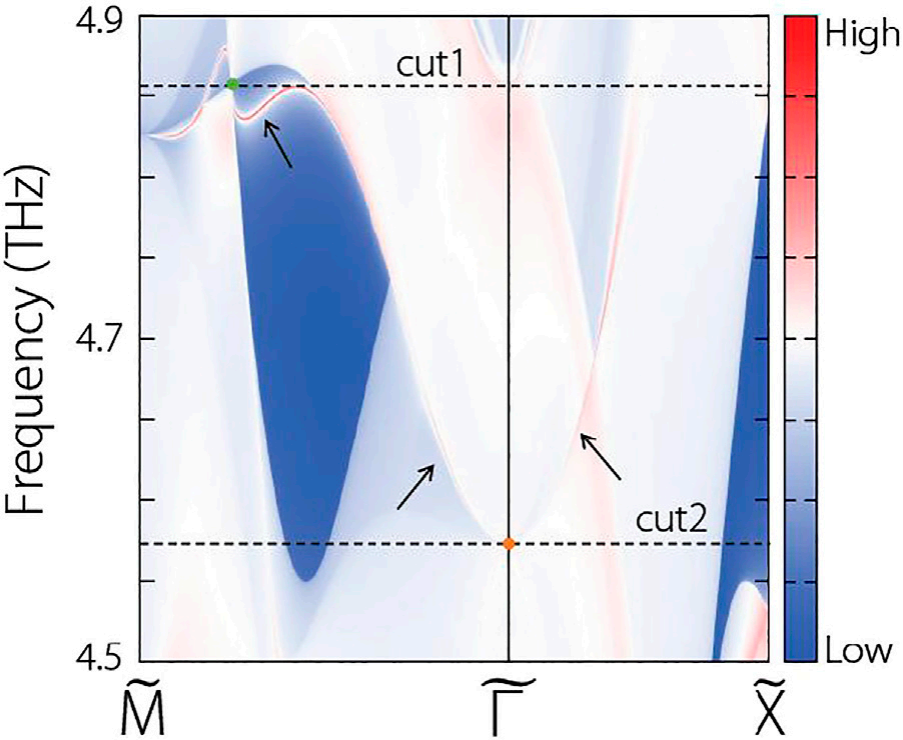
Calculated surface states along the $\tilde{M}-\tilde{\Gamma }-\tilde{X}$ paths of the (001) surface.

In this section, we come to study the project surface states of the [001] NaCl phonons. As shown in Figure 2, we selected some symmetry points, Γ, X and X , and projected these points to $\tilde{\Gamma }$ , $\tilde{X}$ , and $\tilde{M}$ points of the (001) surface. In Figure 5, we collected the results and labeled the positions of the projected TP (green dot) and the projected QCTP (orange dot). One concludes that prominent surface states (Xu et al., 2015; Morali et al., 2019; Li et al., 2020b) connected to the projected TP, which is benefit for experimental detection. Although the surface state connected to the QCTP is a little fuzzy, we can observe its trend and general shape.

**FIGURE 6:**
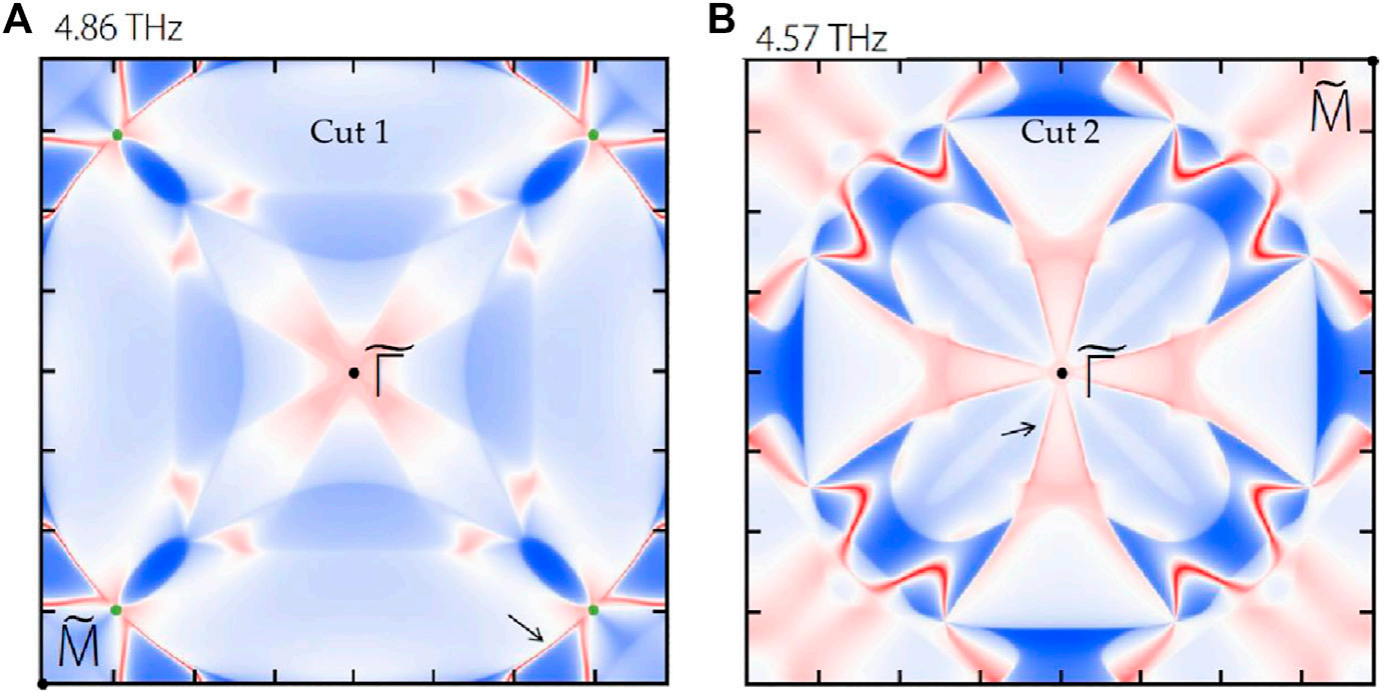
Calculated iso-frequency surface contours at **(A)** 4.86 THz and **(B)** 4.57 THz.

For clarity, we also exhibit the iso-frequency surface contours at 4.86 THz and 4.57 THz in Figure 6A,B, respectively. In Figure 6A, the positions of the projected TP and the connected surface states are marked by a green dot and black arrows, respectively. In Figure 6B, the positions of the projected QCTP and the connected surface states are marked by a black dot and black arrows, respectively. The projected TP/QCTP connected surface states are visible.

## Summary

In this study, we proposed the topological signatures of the NaCl’s phonon dispersion. A systematic theoretical investigation found that this material hosts quadratic and linear nodal lines, TP and QCTP in its phonon dispersion. The QCTP is located at the Γ position, the TP is along the X- Γ , the linear nodal line is along the X- Γ path, and the quadratic nodal line is along the Γ−L. Besides, the surface states are computed and clear surface arc states connected to the projected TP and QCTP can be observed on the (001) surface. Further experimental investigation and verification for these rich topological signatures are expected.

## Data Availability

The original contributions presented in the study are included in the article/Supplementary Material, further inquiries can be directed to the corresponding authors.
